# Evaluation of the Potential Hypoglycemic and Neuroprotective Effects of Rosehip Fruit

**DOI:** 10.3390/molecules31183343

**Published:** 2026-09-21

**Authors:** Fabiola Peña, Felipe González, Emilio Hormazabal, Antonieta Ruiz

**Affiliations:** 1 Departamento de Ciencias Químicas y Recursos Naturales, Scientific and Technological Bioresource Nucleus BIOREN-UFRO, Universidad de La Frontera, Temuco 4811230, Chile; 2 Programa de Doctorado en Ciencias Agroalimentarias y Medioambiente, Facultad de Ciencias Agropecuarias y Medioambiente, Universidad de La Frontera, Temuco 4811230, Chile; 3 Programa de Doctorado en Ciencias Mención Biología Celular y Molecular Aplicada, Facultad de Ciencias Agropecuarias y Medioambiente, Universidad de La Frontera, Temuco 4811230, Chile

**Keywords:** rosehip, alpha-glucosidase, acetylcholinesterase, butyrylcholinesterase

## Abstract

Rosehip fruit (*Rosa* spp.) has sparked growing scientific interest due to its high content of bioactive compounds, which give it significant therapeutic potential. In a context where nutritional issues like obesity and type 2 diabetes continue to increase worldwide, the search for preventive and complementary dietary strategies has become a priority. This study aimed to evaluate the potential hypoglycemic and neuroprotective properties of rosehip fruit (*Rosa canina*, *Rosa moschata*, *Rosa rubiginosa*) through the characterization of its phenolic compounds, evaluation of its antioxidant activity, and analysis of its ability to inhibit alpha-glucosidase, acetylcholinesterase and butyrylcholinesterase enzymatic activities. Four phenolic compounds, mainly quercetin derivatives with high antioxidant activity, were identified. Enzymatic activity revealed significant inhibition of α-glucosidase, with values ranging from 237.9 ± 23.0 μg mL^−1^ to 288.4 ± 22.90 μg mL^−1^, reinforcing the antidiabetic potential of the fruit. However, no inhibitory activity on acetylcholinesterase or butyrylcholinesterase enzymes was detected, indicating limited effectiveness in terms of neuronal protection based on the evaluated criteria. These results suggest that rosehip fruit has significant therapeutic potential in managing diabetes, but limitations in its neuroprotective effect.

## 1. Introduction

*Rosa* spp. also known as rosehip, are perennial species belonging to wild roses [1]. The genus *Rosa* (rosehip) belongs to perennial plants of the family Rosaceae. In modern plant cultivation, wild rose is used as a medicinal, edible, and ornamental plant. Determining species composition and morphological and genetic characteristics is the basis for the rational use of plant resources [2]. In Chile, three species can be found: *Rosa canina* (RC) L., *Rosa rubiginosa* L. (RR), and *Rosa moschata* Herrm. (RM). They are collected between March and June [3,4]. Given its adaptation mechanisms, rosehip is considered a highly expansive species due to its efficient use of light, water, and minerals and its ability to survive in extreme environments [5]. Rosehip fruit has attracted increasing scientific and commercial interest due to the growing consumer demand for functional foods and natural sources of antioxidants and other health-promoting bioactive compounds [6,7].

It is also recognized for its nutritional properties, where a high content of phenolic compounds, flavonols, ascorbic acid, carotenoids, organic acids, tocopherols, and antioxidant activity have been reported [4,6,8].

These compounds have attracted considerable interest as natural sources of antioxidants because they may help modulate redox balance, neutralize reactive species, and attenuate part of the damage associated with oxidative stress; however, their clinical efficacy depends on the specific compound, dose, bioavailability, and the context of use [9,10,11].

In 2024, 589 million adults (20–79 years) worldwide had diabetes, and this number is expected to increase to 643 million in 2030 and 783 million in 2045. Diabetes was responsible for 3.4 million deaths in 2024 [12]. However, other forms of diabetes also exist, including gestational diabetes mellitus, monogenic diabetes, and specific types resulting from other causes, such as diseases of the exocrine pancreas or drug-induced diabetes, although these are considerably less common than type 1 and type 2 [13].

Nutrition and diet therapy are fundamental strategies in the management of diabetes mellitus [14]. These approaches not only help healthcare professionals and patients control the disease but also contribute to preventing the onset of its associated complications [13,15]. Among these strategies, lifestyle modifications, particularly shifting toward healthier eating habits and adopting recommended dietary patterns, are key measures to curb the increasing prevalence of diabetes mellitus [16]. Currently, it is believed that bioactive compounds from food can aid in the alleviation of diabesity, thanks to their low toxicity and the fact that they do not exhibit significant adverse effects like conventional drugs [17].

Human life expectancy has increased in recent years, attributable to medical advances and higher living standards, resulting in a significant rise in the elderly population and a rapid progression in the overall pace of population aging [18]. There is an increased, associated prevalence of neurodegenerative disorders, characterized by the gradual decline of neuronal structure and functionality, resulting in the death of neurons, which deteriorates over time and eventually leads to nervous system dysfunction [19]. Diseases such as Alzheimer’s, Parkinson’s, Huntington’s, amyotrophic lateral sclerosis, and spinocerebellar ataxia exemplify this group of conditions [17].

Rosehip fruit has a wide variety of bioactive properties, such as antioxidant, anti-inflammatory, anticancer, and anti-inflammatory capacities [4,20,21,22]. The bioactive compounds of rosehip, including flavonoids, triterpenoids, and phytosterols, can modulate multiple inflammatory pathways by inhibiting pro-inflammatory enzymes, reducing cytokine and chemokine production, and attenuating oxidative stress, thereby suppressing inflammatory responses [7,23].

Both the antioxidant and anti-inflammatory effects of rosehip coincide with its clinical response according to studies conducted using it as a pharmacology of osteoarthritis. This is associated with compounds such as phenolics, terpenoids, galactolipids, carotenoids, fruit acids, and fatty oils [24]. Furthermore, recent research has shown that rosehip extracts possess significant neuroprotective effects, attributed primarily to their ability to modulate oxidative stress, reduce neuroinflammation, and preserve the structural integrity of neurons [25]. In vitro studies have shown that phenolic extracts of *Rosa canina* protect neuronal cells from oxidative damage, promoting cell viability and supporting their neuroprotective activity in models of cerebral ischemia [26]. Similarly, it has been reported that the compounds present in rosehip fruit improve memory and cognitive parameters in animal models, supporting their potential use in the prevention of neurodegenerative disorders like Alzheimer’s disease [27].

Building on this background, it is hypothesized that rosehip fruit contains bioactive compounds with potential hypoglycemic and neuroprotective effects that may positively influence glucose control and protect neuronal cells from damage associated with neurodegenerative diseases. The general objective of this study was to determine the potential hypoglycemic and neuroprotective properties of rosehip fruit by characterizing its phenolic compounds, evaluating its antioxidant activity, and analyzing its ability to inhibit the enzymes alpha-glucosidase, acetylcholinesterase, and butyrylcholinesterase as indicators of its potential properties in the control of diabetes and neuronal protection.

## 2. Results

### 2.1. Profiles and Concentrations of Phenolic Compounds and Total Phenols

Four phenolic compounds were detected in *R. canina* (RC), *R*. *rubiginosa* (RR), and *R. moschiata* (RM) extracts, corresponding to quercetin–hexoside isomers, quercetin–rhamnoside, and an unidentified compound (tentatively quercetin-derivative), all from the flavonol family, according to mass spectrometry and UV-vis spectral data (Table 1).

**Figure 1:**
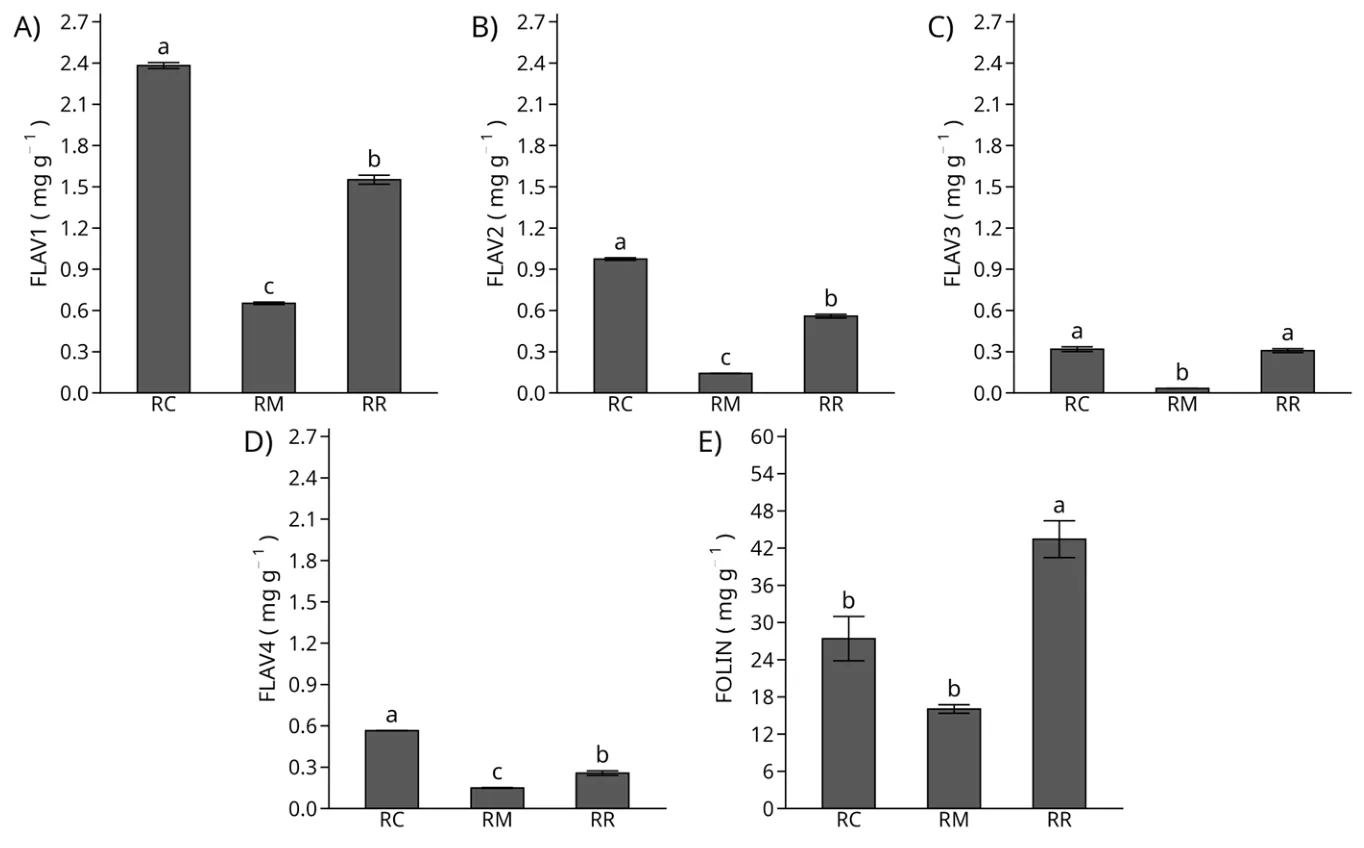
Individual and total phenolic concentrations in rosehip fruit. Three species of rosehip were evaluated, *Rosa canina* (RC), *Rosa moschata* (RM), and *Rosa rubiginosa* (RR). Flavonol concentrations were grouped into four categories: (**A**) flavonol 1, (**B**) flavonol 2, (**C**) flavonol 3, (**D**) flavonol 4, and (**E**) total phenols determined by the Folin–Ciocâlteu method. The flavonol groups corresponded to the following compounds: FLAV 1: quercetin–hexoside 1, FLAV 2: quercetin–hexoside 2, FLAV 3: unidentified flavonol, and FLAV 4: quercetin–rhamnoside. Different letters in each sub-figure indicate the presence of statistically significant differences according to Tukey’s multiple range test (*p* ≤ 0.05).

Regarding the concentration of individual phenolic compounds determined by HPLC-DAD, a consistent trend was observed in the concentrations of individual compounds as well as in total phenolic content. In all cases, the highest concentrations were detected in RC fruit, whereas the lowest concentrations were found in RM fruit. Regarding individual compounds, the highest concentrations were detected in quercetin–hexoside 1, with values ranging from 0.65 ± 0.01 mg g^−1^ in RM and 2.38 ± 0.01 mg g^−1^ in RC (Figure 1A). Quercetin–hexoside 2 concentrations ranged from 0.14 ± 0.00 mg g^−1^ (RM) to 0.97 ± 0.01 mg g^−1^ (RC) (Figure 1B). Values were considerably lower for the unidentified compound, ranging from 0.03 ± 0.00 mg g^−1^ (RM) to 0.32 ± 0.01 mg g^−1^ (RC) (Figure 1C), while in quercetin–rhamnoside, concentrations ranged from 0.15 ± 0.01 mg g^−1^ in RM to 0.56 ± 0.00 mg g^−1^ in RC (Figure 1D). Total phenolic content quantified using the Folin method ranged from 16.06 ± 0.50 in RM to 43.44 ± 1.55 in RR (Figure 1E).

### 2.2. Antioxidant Activity

**Figure 2:**
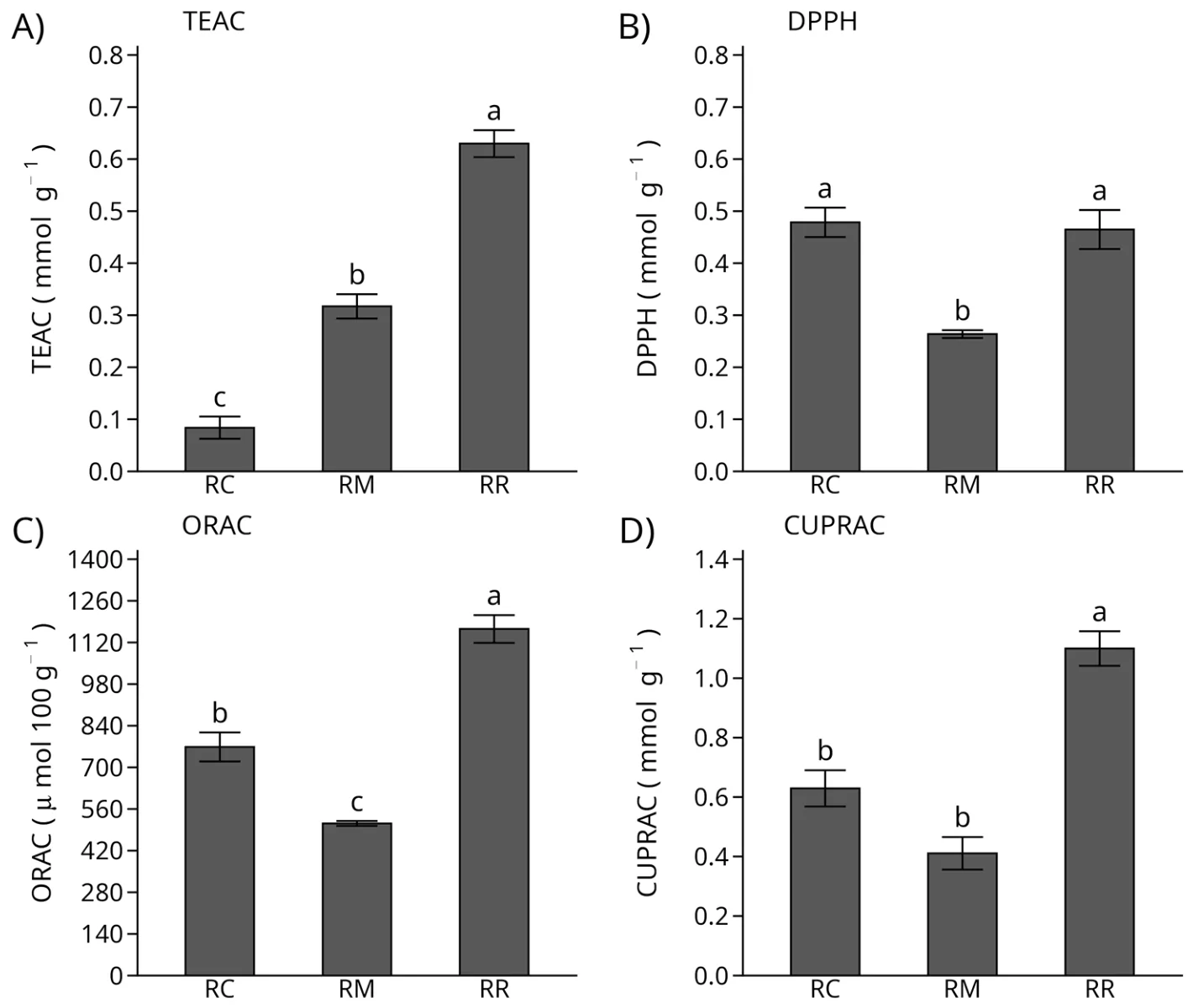
Antioxidant activities by spectrophotometric methods. Three varieties of rosehip were evaluated: *Rosa canina* (RC), *Rosa moschata* (RM), and *Rosa rubiginosa* (RR). (**A**) Antioxidant activity by Trolox equivalent antioxidant capacity method (TEAC). (**B**) Antioxidant activity by 2,2-diphenyl radical (DPPH) method. (**C**) Antioxidant activity by oxygen radical antioxidant capacity (ORAC) method. (**D**) Antioxidant activity by cupric reducing antioxidant capacity (CUPRAC) method. Different letters in each sub-figure indicate the presence of statistically significant differences according to Tukey’s multiple range test. (*p* ≤ 0.05).

The antioxidant activity determined by the TEAC, DPPH, CUPRAC and ORAC methods showed notable differences and different trends between the RC, RM, and RR samples. In the TEAC assay, values ranged from 0.08 ± 0.01 mmol g^−1^ (RC) to 0.63 ± 0.01 mmol g^−1^ (RR), with the latter being the sample with the highest antioxidant capacity according to this method (Figure 2A). In DPPH, levels ranged from 0.26 ± 0.01 mmol g^−1^ (RM) to 0.48 ± 0.02 mmol g^−1^ (RC), with similar values in RR (0.46 ± 0.02 mmol g^−1^), with no statistically significant differences between the latter two (Figure 2B). In the ORAC assay, the results ranged from 511.5 ± 19.9 μmol/100 g (RM) to 1165.4 ± 26.9 μmol/100 g (RR), while RC presented an intermediate value (768.7 ± 26.3 μmol/100 g) (Figure 2C). Finally, with the CUPRAC method, the values ranged between 0.41 ± 0.03 mmol g^−1^ (RM) and 1.10 ± 0.04 mmol g^−1^ (RR), again highlighting RR as the sample with the highest antioxidant activity (Figure 2D). Overall, the RR sample consistently presented the highest antioxidant activity values in the TEAC, CUPRAC, and ORAC methods, suggesting a greater free radical neutralization capacity. In contrast, with the DPPH method, RC and RR showed similar values, with no statistically significant differences between them, although higher than those of RM.

### 2.3. Biological Activities

**Figure 3:**
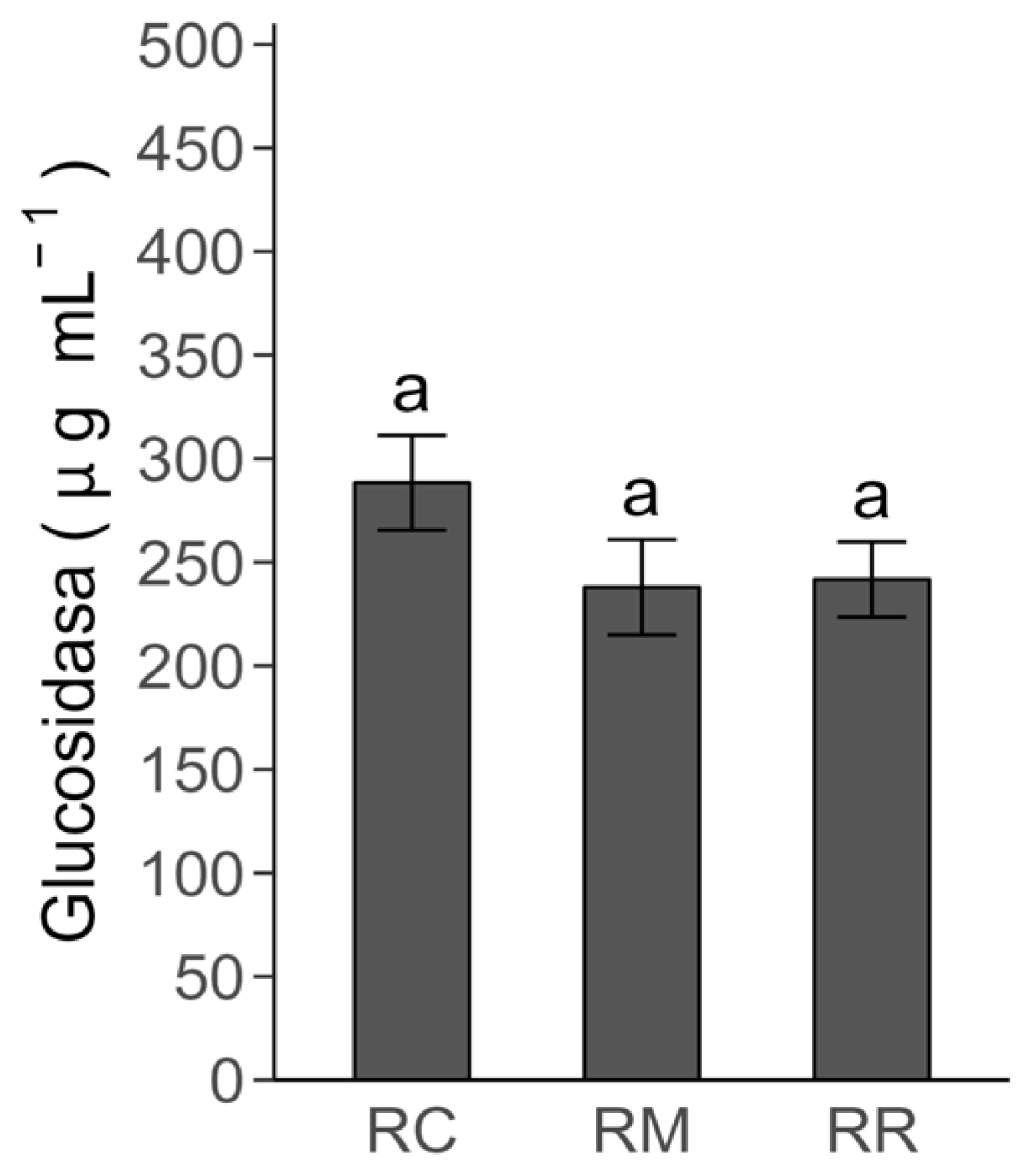
Inhibition of α-glucosidase of extracts by three rosehip varieties: *Rosa canina* (RC), *Rosa moschata* (RM), and *Rosa rubiginosa* (RR). Different letters in each sub-figure indicate the presence of statistically significant differences according to Tukey’s multiple range test (*p* ≤ 0.05).

In the determination of the inhibition of α-glucosidase, all extracts showed activities, with values for RC of 288.4 ± 22.9 μg mL^−1^, for RM 237.9 ± 23.0 μg mL^−1^ and for RR 241.7 ± 18.1 μg mL^−1^. No statistical differences were observed between the samples (Figure 3).

Regarding acetylcholinesterase inhibitory activity, the samples were evaluated at concentrations ranging from 100 to 2000 µg mL^−1^ and did not show any inhibition of acetylcholinesterase in these ranges (Table 2). Therefore, it was decided to evaluate them at higher concentrations of 2000 to 6000 µg mL^−1^. The results showed inhibition, but none of the samples reached 50% inhibition at the maximum concentration evaluated. The control used, galantamine, had an IC50 of 0.447 µg mL^−1^ (1.56 µM), a value within the published range.

In the case of butyrylcholinesterase inhibitory activity, samples were evaluated within a concentration range of 2000–6000 µg mL^−1^, considering the results obtained for acetylcholinesterase inhibition (Table 3). The extracts showed no inhibition at the highest concentration tested (6000 µg mL^−1^). The control used, galantamine, had an IC50 of 9.45 µg mL^−1^ (32.87 µM), a value within the published range.

### 2.4. Global Analysis

**Figure 4:**
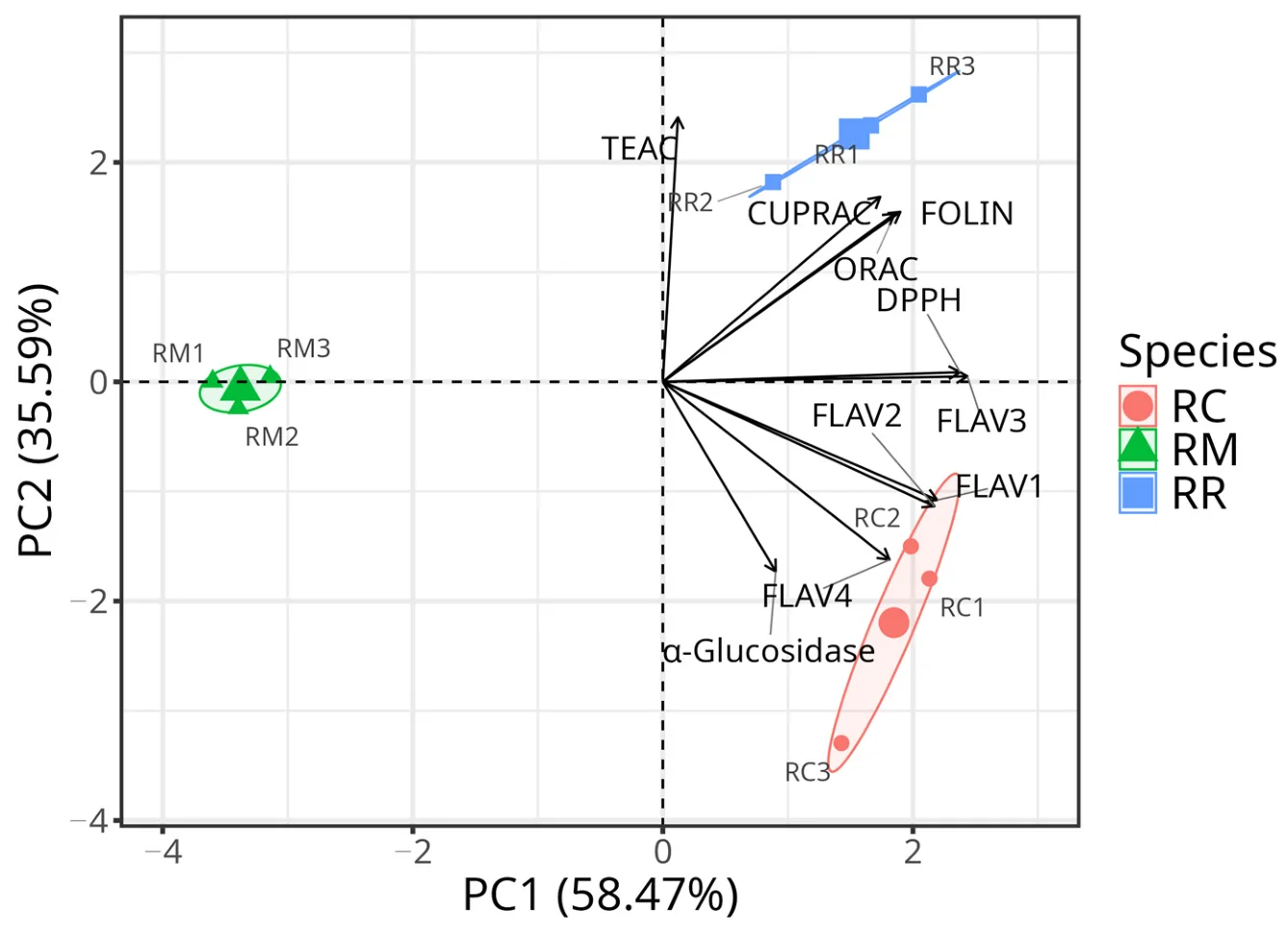
Principal component analysis (PCA) results representing the *Rosa canina* (RC), *Rosa moschata* (RM), and *Rosa rubiginosa* (RR) treatments. The nomenclature of the variables are as follows: antioxidant activity by Trolox equivalent antioxidant capacity method (TEAC), antioxidant activity by 2,2-diphenyl radical (DPPH) method, antioxidant activity by cupric reducing antioxidant capacity (CUPRAC) method, antioxidant activity by oxygen radical antioxidant capacity (ORAC) method, and total phenols by Folin–Ciocâlteu (FOLIN) method. FLAV 1: flavonol 1, FLAV 2: flavonol 2, FLAV 3: flavonol 3, FLAV 4: flavonol 4. α-Glucosidase: α-glucosidase.

The principal component analysis (PCA) (Figure 4) explained 94.06% of the total variance, with PC1 accounting for 58.47% and PC2 for 35.59%. According to the PCA, antioxidant variables are those that most influence principal component 2 (PC2), while phenolic compounds and glucosidase mainly influence principal component 1 (PC1). The RC samples are positively associated with the flavonoid compounds FLAV1, FLAV2, and FLAV4, as well as with the activity of the glucosidase enzymatic activity, suggesting a greater accumulation of phenolic compounds in these species. On the other hand, the RR samples show a close relationship with the variables associated with antioxidant capacity (CUPRAC, ORAC, TEAC) and total phenolic compounds (Folin), indicating greater antioxidant potential. In contrast, the RM treatment is located in an intermediate region of the component space without a clear association with the evaluated variables, suggesting a less marked or intermediate biochemical profile. The neuroprotective effects were not considered in these analyses.

## 3. Discussion

It is remarkable that among all the evaluated extracts, the RC sample consistently exhibited the highest flavonol concentrations, followed by RR, while RM showed the lowest values in each case. Furthermore, statistically significant differences were noted in the three quercetin derivative concentrations, suggesting a differential distribution of phenolic compounds among the fractions. The identification of phenolic compounds in this study showed a reduced number of metabolites compared to those reported in the literature. Peña et al. [4] identified an anthocyanin (cyanidin-3-glucoside), a flavan-3-ol (catechin), a hydroxycinnamic acid (galloylquinic acid), and six flavonols, mainly glycosylated derivatives of quercetin, in rosehip fruit. In a broader study, a total of 46 phenolic compounds in *Rosa canina* fruit were characterized, including cyanidin-3-glucoside as a representative anthocyanin, and flavan-3-ols and proanthocyanidins as the most abundant groups, in addition to simple phenolic acids, flavanones, flavonols, flavanones, and the dihydrochalcone phloridzin [28]. The low concentration of phenolic compounds detected in this study could be attributed in part to the degradation of these metabolites during the processing and storage of the extract. On the other hand, the stability of rosehip juice compounds and phenolic compounds, including flavonoids and phenolic acids, is highly susceptible to factors such as temperature, light exposure, pH, and storage time [29]. These findings explain the possible degradation of the compounds present in our extracts, since they were exposed to some of these factors during their preparation.

In this present study, it is remarkable that *Rosa canina* species stood out for presenting the highest concentrations of individual phenolic compounds, while *Rosa moschata* showed the lowest levels. In particular, it was observed that quercetin derivatives, such as quercetin–hexoside isomers and quercetin–rhamnoside, were the predominant compounds. This trend aligns with previous studies that identified these flavonols as phenolic compounds in *R. rubiginosa* fruit [30]. The variability in the concentration of these compounds can be attributed to genetic and environmental factors, climatic conditions, and fruit ripening stage [31]. Furthermore, the presence of an unidentified compound at lower concentrations suggests the existence of other phenolic metabolites that could contribute to the biological activity of this fruit and that merit more detailed characterization.

The results obtained show significant differences in antioxidant activity among the samples analyzed, with RR consistently presenting the highest values in all methods. This coincides with the total phenol concentration results and suggests an important free radical neutralization capacity, likely due to a higher concentration of phenolic compounds including flavonoids with multiple antioxidant mechanisms [32].

The results revealed significant antioxidant activity among the rosehip samples, with the RR sample showing the highest values in the TEAC, CUPRAC, and ORAC assays. In the TEAC assay, RR exhibited an antioxidant capacity of 0.63 ± 0.01 mmol g^−1^. This value falls within the wide range reported for raspberry (0.0017–1.003 mmol g^−1^) [33,34] and blueberry (0.0033 to 0.327 mmol g^−1^) [35,36], representing relevant antioxidant activity. Similarly, the highest DPPH value was observed in the RC sample (0.48 ± 0.02 mmol g^−1^). Although this value falls within the range reported for raspberry (0.0014–0.85 mmol g^−1^), comparisons with blueberry must be interpreted with caution because antioxidant capacity is strongly influenced by extraction procedures, sample composition, assay conditions, and the basis used to express the results. In the CUPRAC assay, RR showed a value of 1.10 ± 0.04 mmol g^−1^. After conversion to a common unit (1100 ± 40 µmol TE g^−1^), this value cannot be directly compared with the blueberry range reported as 15.71–36.89 µmol TE g^−1^ without verifying whether the sample basis and analytical conditions were equivalent [37]. In the ORAC assay, RR showed a value of 1165.40 ± 26.88 µmol/100 g, which is within the wide range reported for raspberry (82.64–4790 µmol/100 g) [38,39], but below the range reported for blueberry (2050–6747 µmol/100 g) [40,41]. Overall, these comparisons indicate that the rosehip samples exhibited substantial antioxidant activity; However, differences in analytical methods, extraction conditions, sample matrices, and units of expression limit direct comparisons between fruit species.

Taken together, these results indicate that although the absolute antioxidant activity values of rosehip may be lower than those of fruit widely recognized for their high antioxidant potential, such as raspberry and blueberry, this wild species still presents a significant capacity to neutralize free radicals. The high antioxidant capacity of RR could be related to relevant biological effects, such as protection against cellular oxidative damage, and supports its potential in functional, nutraceutical, or therapeutic applications, especially considering the background highlighting the neuroprotective and antidiabetic activity of *Rosa canina* extracts [20,42].

Although *Rosa canina* exhibited higher concentrations of individual flavonols, particularly quercetin derivatives, the total phenolic content (Folin–Ciocâlteu) and antioxidant activity were consistently higher in *R. rubiginosa*. This apparent discrepancy may be explained by the broader diversity and synergistic interactions of phenolic and non-phenolic compounds present in *R. rubiginosa*. The Folin–Ciocâlteu method is not entirely specific to flavonols, but rather detects a wide range of reducing substances, including other phenolics, ascorbic acid, and interfering compounds [32], which may be present at higher levels in *R. rubiginosa*. Furthermore, antioxidant activity depends not only on the concentration of individual compounds but also on their structural characteristics and possible synergistic effects between different metabolites [8,43]. Compounds such as phenolic acids, flavonoids, carotenoids, tocopherols, and vitamin C, which are widely reported in rosehip fruit, are considered the main contributors to its antioxidant capacity through complementary and synergistic mechanisms [6,7].

Therefore, despite *R. canina* having higher levels of specific flavonols, the more complex phytochemical profile of *R. rubiginosa* likely explains its superior total phenolic content and antioxidant activity.

This study demonstrated that *R. canina* extracts have an inhibitory capacity on the α-glucosidase enzyme. Although previous studies have already reported hypoglycemic effects associated with rosehip consumption, two recent reviews highlight that its therapeutic benefits go beyond glycemic control [7,44,45].

Our results were compared with the inhibitory activity against α-glucosidase of different compounds and extracts. Honokiol, a natural compound from *Magnolia obovata* Thunb., showed a concentration-dependent inhibition, with an IC_50_ value of 84.45 μg/mL, indicating moderate inhibitory activity [46]. The results obtained show that unlike other plant extracts reported in the literature, such as *Jacaranda* Juss., the extracts evaluated in this study do not present a significant inhibition of acetylcholinesterase when applied at conventional concentrations [47].

The results associated with butyrylcholinesterase suggest either an absence or very low concentration of compounds capable of interacting with the enzyme, or a weak structural affinity of the metabolites present for the active site of BChE. Compared with other studies that have reported significant inhibition of this enzyme with plant extracts from *Salvia* (Lamiaceae) rich in flavonoids and alkaloids [48], the results obtained here indicate a limited cholinergic profile.

In this study, *R. canina* fruit extracts did not show significant inhibitory activity on cholinesterase enzymes, specifically acetylcholinesterase and butyrylcholinesterase. This result contrasts with various reports in the literature that have associated the *Rosa* genus with neuroprotective properties attributed to its phytochemical composition, particularly the presence of flavonoids, phenolic acids, tannins, and other polyphenols [49]. Therefore, although our results do not show cholinergic enzyme inhibition, they suggest *Rosa canina* may have neuroprotective potential. Rather, they suggest the need for additional studies addressing other pathways of action.

Our results observed in the PCA are consistent with those reported by other authors, who have documented high antioxidant activity and flavonol content in extracts from different species of the genus *Rosa* [4]. Flavonols have been widely studied due to their bioactive properties, and several individual structures have been shown to exert beneficial effects on human health [43]. In addition to their recognized capacity as antioxidants, flavonols also function as enzyme modulators, which could explain the observed association between these compounds and glucosidase activity [43]. Overall, the results obtained demonstrate the phytochemical richness of rosehip fruit, particularly in phenolic compounds and flavonols, which not only contribute to its outstanding antioxidant capacity but also to its modulatory action on enzymes. This highlights the therapeutic potential of the fruit and the relevance of its functional properties.

## 4. Materials and Methods

### 4.1. Reagents

Methanol, water, acetonitrile (HPLC grade), Folin–Ciocâlteu reagent, ethanol, and formic acid were obtained from Merck (Darmstadt, Germany). Phosphoric acid, Trolox (6-hydroxy-2,5,7,8 acid tetramethylchroman-2-carboxylic acid) (97% purity), ABTS (2,20-azino-bis (3-ethylbenzothiazoline-6-sulfonic acid)) (>98% purity), DPPH (2,2-diphenyl-1-picrylhydrazyl), AAPH (2,2′-azobis 2-methyl-propionamidine dihydrochloride) neocuproine (≥98), fluorescein sodium salt, α-glucosidase, p-nitrophenyl-α-D-glucopyranoside, sodium phosphate buffer, Tris solution, 5,5′-dithiobis-(2-nitrobenzoic acid (DTNB) and acetylthiocholine iodide (ATCI) were obtained from Sigma-Aldrich (Steinheim, Germany).

### 4.2. Sample Preparation

Rosehip fruit was collected in March 2023 in Nueva Imperial 38°45′09.5″ S 73°04′21.9″ W, Araucanía Region, Chile. Three species, *Rosa canina*, *Rosa moschata*, and *Rosa rubiginosa*, were identified, collected, and stored at −80° to be subsequently freeze-dried.

### 4.3. Determination of Phenolic Compounds

The extract was prepared based on what was reported by Bravo et al. [50] with some modifications. Initially, 10 g of freeze-dried rosehip fruit was weighed, and 30 mL of methanol was added. Sonication was applied with an ultrasound bar (Sonics & Materials Inc, Newtown, USA) for 60 s at an amplitude of 40%. The samples were shaken for 10 min at 200 rpm on a shaker (Zhicheng, Shangai, China) and centrifuged for 10 min at 4000 rpm. The extract was rotovaporated and resuspended in HPLC water. It was then stored at −80° for 48 h to then be freeze-dried. Finally, 10 mg of the lyophilized extract were resuspended in 1 mL of methanol and used for the analysis.

Qualitative and quantitative analyses were performed using high-performance liquid chromatography (HPLC-DAD), as reported by Peña et al. [4]. An HPLC system (Shimadzu, Tokyo, Japan) equipped with an LC-20AT quaternary pump, a CTO-oven 20A, a SIL-20, a DGU-20A5R degassing unit, and an SPD-M20A UV-visible diode array spectrophotometer was used. Instrument control and data collection were performed using Lab Solutions 5.96 software (Shimadzu, Duisburg, Germany). A Kromasil C_18_ column (100 × 4.6 mm, 2.5 μm) (Bohus, Sweeden) and a Waters NovaPak C_18_ guard column (Taunton, USA) (22 × 3.9 mm, 4 µm) were used. Identity assignments were performed according to the methodology described in the literature [51] using an HPLC-DAD-QTOF-MS/MS Compact system (Bruker Daltonics GmbH, Bremen, Germany). Instrument control and data collection were conducted using Compass DataAnalysis 4.4 SR1 software (Bruker Daltonics GmbH, Bremen, Germany).

The total phenolic content in the fruit extract was measured by the Folin–Ciocâlteu colorimetric procedure adapted to microplates, as reported by Bravo et al. [50]. Different reagents were added to 1.5 mL Eppendorf tubes, then shaken and incubated for 30 min in the dark. Finally, the reading was carried out in 96-well microplates at an absorbance of 750 nm using a Synergy HTX reader (BioTek Instruments, Winooski, VT, USA). The results are expressed as gallic acid equivalent (GAE).

### 4.4. Determination of Antioxidant Activity

The antioxidant activity was determined as reported by Peña et al. [4] using the cupric reducing antioxidant capacity (CUPRAC) method, the Trolox equivalent antioxidant capacity (TEAC) method, the 2,2-diphenyl radical method, and the 2,2-diphenyl-1-picrylhydrazyl (DPPH) method, adapted to 96-well plates (Synergy HTX, BioTek Instruments, Winooski, VT, USA). The oxygen radical absorbance capacity (ORAC) was assessed based on reports in the literature [52]. Trolox was used as a standard in all methods, and results are expressed as Trolox equivalent (TE).

### 4.5. Determination of α-Glucosidase Inhibition

α-Glucosidase inhibition was determined based on Proenca et al. [53]. The inhibitory activity of the extracts against α-glucosidase was assessed using a 96-well microplate. This was then incubated for 10 min at 37 °C in an orbital shaker (Zhicheng, Shangai, China). The enzyme solution was prepared at a concentration of 0.15 U mL^−1^ in sodium phosphate buffer (100 mM, pH 6.9, containing 0.006 M NaCl). The reaction mixture consisted of 100 μL of sodium phosphate buffer (100 mM, pH 6.9), 75 μL of p-nitrophenyl-α-D-glucopyranoside (PNP, 1.2 mM) as substrate, and 25 μL of the extracts at concentrations of 25, 50, 100, 250, 500, and 1000 μg mL^−1^. The mixtures were incubated for 10 min at 37 °C in a orbital shaker. Subsequently, 50 μL of the α-glucosidase solution was added and the mixtures were further incubated for 15 min at 37 °C. The reaction was stopped by adding 50 μL of Tris solution (0.5 M). The absorbance of the released p-nitrophenol was measured at 405 nm using a BioBase-EL10A microplate reader (Jinan, Shandong, China). Acarbose was used as a positive inhibition control at concentrations ranging from 100 to 2000 μg mL^−1^. All measurements were performed in triplicate, and the results are expressed based on the sample concentration required to inhibit 50% of the total enzyme activity (IC_50_).

### 4.6. Acetylcholinesterase Inhibitory Activity

The inhibition of acetylcholinesterase (AChE) by the compounds was determined using the spectrophotometric method described by Ellman et al. [54], with modifications according to Mella et al. [55]. Initially, 50 µL of AChE (0.50 U/mL) and 50 µL of the sample in buffer solution were mixed. The plates were incubated for 30 min at 25 °C. Subsequently, 100 µL of a substrate solution composed of DTNB and ATCI was added. After 3 min, the absorbance (Abs) was measured at 405 nm using a microplate reader. Activity was calculated using the following formula:

$$\%\;inhibition=\left(1-\left(\frac{{Abs}_{sample}-{Abs}_{control\;sample}}{{Abs}_{enzymatic\;reaction}-{Abs}_{control\;enzymatic\;reaction}}\right)\right)\times 100$$

### 4.7. Butyrylcholinesterase Inhibitory Activity

Butyrylcholinesterase (BuChE) inhibition by isolated compounds was determined by the spectrophotometric method according to Ellman et al. [54]. Initially, 50 µL of BuChE in phosphate-buffered saline and 50 µL of the samples dissolved in the same buffer was added to the wells. The plates were incubated for 30 min at 25 °C before adding 100 µL of the substrate solution in HPLC-grade water. After 5 min, the absorbance at 405 nm was read in a BIOBASE-EL10A microplate reader (Jinan, Shandong, China). Enzyme inhibition was calculated as a percentage compared to a control using a buffer without inhibitor. IC50 values are the means ± SD of three determinations. Galantamine hydrobromide was used as a positive control.

### 4.8. Statistical Analysis

All statistical analyses and figure generation were performed using R software (version 4.2.1). For each species, three independently processed extracts were considered experimental units (*n* = 3 per species). Each extract was measured in triplicate, and these technical replicates were averaged prior to statistical analysis; therefore, statistical tests were performed using the extract means rather than individual technical measurements. Normality and homogeneity of variances were assessed using the Shapiro–Wilk and Levene tests, respectively. Once these assumptions were verified, differences among species were evaluated using one-way analysis of variance (ANOVA), followed by Tukey’s honest significant difference (HSD) test when significant effects were detected (*p* < 0.05). These analyses were performed using the “agricolae” package (v1.3.5). Results are presented as means ± standard error (SE), calculated from the three independent extracts, and different letters indicate statistically significant differences among species.

For principal component analysis (PCA), the mean value of the technical replicates for each extract was used, resulting in nine experimental units (three extracts per species). Variables were standardized by z-score normalization (mean-centered and scaled to unit variance) before PCA to ensure equal contribution regardless of measurement scale. Variable loadings and the proportion of total variance explained by each principal component were determined. The PCA score plot was generated from the nine independent experimental units, and 95% confidence regions around the centroid of each species were calculated using the “FactoMineR” (v2.7) and “factoextra” (v1.0.7) packages.

## 5. Conclusions

The results of this study indicate that rosehip fruit has the ability to inhibit alpha-glucosidase activity, which could contribute potentially to the regulation of blood glucose levels. The identification of phenolic compounds and high antioxidant activity reinforce the idea that the fruit may offer benefits in mitigating oxidative damage associated with metabolic diseases. However, the lack of inhibitory activity on the enzymes acetylcholinesterase and butyrylcholinesterase suggests that the neuroprotective potential of rosehip fruit may be limited or null under the conditions evaluated. These findings suggest that although rosehip fruit has promising potential as an antidiabetic agent, its ability to offer neuronal protection warrants further investigation.

## Figures and Tables

**Table 1:** Identification of phenolic compounds in rosehip extracts by HPLC-DAD-ESI-MS/MS.

Peak Number	t_R_ (min)	Abbreviation	Tentative Identification	[M − H]^−^	Product Ions	٨_max_ (nm)
1	17.3	FLAV1	Quercetin–hexoside 1	463.1	301.1	353
2	18.1	FLAV2	Quercetin–hexoside 2	463.1	300.0	353
3	20.2	FLAV3	Quercetin derivative	615.1	300.0	347
4	21.3	FLAV4	Quercetin–rhamnoside	447.1	300.0	346

**Table 2:** Results of inhibitory activity of extracts on acetylcholinesterase activity. Percentage enzyme inhibition at 6000 µg/mL and IC_50_ (µg mL^−1^) of extracts from three rosehip species—*Rosa canina* (RC), *Rosa moschata* (RM), and *Rosa rubiginosa* (RR)—compared to the standard inhibitor galantamine. IC_50_ values greater than 6000 µg mL^−1^ indicate low inhibitory activity.

Sample	Inhibition % (6000 µg mL^−1^)	IC50 (µg mL^−1^)
RR	7.2	>6000
RC	26.24	>6000
RM	22.46	>6000
Galantamine	-	0.447

**Table 3:** Inhibitory activity of rosehip extracts in butyrylcholinesterase activity. Percentage enzyme inhibition at 6000 µg mL^−1^ and IC_50_ (µg mL^−1^) of extracts from three rosehip species—*R. canina* (RC), *R. moschata* (RM) and *Rosa rubiginosa* (RR)—compared to the standard inhibitor galantamine. IC_50_ values greater than 6000 µg mL^−1^ indicate low inhibitory activity.

Sample	Inhibition % (6000 µg mL^−1^)	IC50 (µg mL^−1^)
RR	-	>6000
RC	-	>6000
RM	-	>6000
Galantamine	-	9.45

## Data Availability

The data presented in this study are available upon request from the corresponding author.
